# A new model of portal vein thrombosis in rats with cirrhosis induced by partial portal vein ligation plus carbon tetrachloride and intervened with rivaroxaban

**DOI:** 10.1186/s12876-024-03253-4

**Published:** 2024-05-13

**Authors:** Yanan Guo, Sisi Dong, Meng Li, Yanyan Tao, Jing Lv, Chenghai Liu

**Affiliations:** 1 Institute of Liver Diseases, Shuguang Hospital Affiliated to Shanghai University of Traditional Chinese Medicine, Shanghai, 201203 China; 2 Shanghai Key Laboratory of Traditional Chinese Clinical Medicine, Shanghai, China; 3 Key Laboratory of Liver and Kidney Diseases, Ministry of Education, Shanghai, China; 4 Department of Liver Disease, Shuguang Hospital Affiliated to Shanghai University of Traditional Chinese Medicine, Shanghai, 201203 China; 5 Liuhe District Hospital of Traditional Chinese Medicine, Nanjing, Jiangsu Province China

**Keywords:** Portal vein thrombosis, Cirrhosis, Partial portal vein ligation, Carbon tetrachloride, Model, Rivaroxaban, Mechanism

## Abstract

**Background and aims:**

Portal vein thrombosis (PVT) is a common complication of liver cirrhosis that can aggravate portal hypertension. However, there are features of both PVT and cirrhosis that are not recapitulated in most current animal models. In this study, we aimed to establish a stable animal model of PVT and cirrhosis, intervene with anticoagulant, and explore the related mechanism.

**Methods:**

First, 49 male SD rats received partial portal vein ligation (PPVL), and 44 survival rats were divided into 6 groups: PPVL control group; 4-week, 6 -week, 8-week, and 10-week model group; and the rivaroxaban (RIVA)-treated group. The rats were intoxicated with or without carbon tetrachloride (CCl_4_) for 4–10 weeks. Seven normal rats were used as the normal controls. Serum alanine aminotransferase (ALT) and aspartate aminotransferase (AST) levels and parameters for blood coagulation were all assayed with kits. Liver inflammation, collagen deposition and hydroxyproline (Hyp) levels were also measured. The extrahepatic macro-PVT was observed via portal vein HE staining, etc. The intrahepatic microthrombi was stained via fibrin immunohistochemistry. The portal blood flow velocity (PBFV) and diameter were detected via color Doppler ultrasound. Vascular endothelial injury was evaluated by von Willebrand Factor (vWF) immunofluorescence. Fibrinolytic activity was estimated by western blot analysis of fibrin and plasminogen activator inhibitor-1 (PAI-1).

**Results:**

After PPVL surgery and 10 weeks of CCl_4_ intoxication, a rat model that exhibited characteristics of both cirrhosis and extra and intrahepatic thrombi was established. In cirrhotic rats with PVT, the PBFV decreased, both factors of pro- and anti-coagulation decreased, but with relative hypercoagulable state, vascular endothelial injured, and fibrinolytic activity decreased. RIVA-treated rats had improved coagulation function, increased PBFV and attenuated thrombi. This effect was related to the improvements in endothelial injury and fibrinolytic activity.

**Conclusions:**

A new rat model of PVT with cirrhosis was established through partial portal vein ligation plus CCl_4_ intoxication, with the characteristics of macrothrombi at portal veins and microthrombi in hepatic sinusoids, as well as liver cirrhosis. Rivaroxaban could attenuate PVT in cirrhosis in the model rats. The underlying mechanisms of PVT formation in the rat model and pharmacological action of rivaroxaban are related to the regulation of portal blood flow, coagulant factors, and vascular endothelial cell function.

**Supplementary Information:**

The online version contains supplementary material available at 10.1186/s12876-024-03253-4.

## Introduction

Portal vein thrombosis (PVT), defined as the presence of thrombi in the lumen of the main portal vein that can extend into intrahepatic or extrahepatic venous branches [1], is a common complication in patients with cirrhosis. As liver function deteriorates, the incidence of PVT complications in cirrhotic patients increases. Moreover, the development of PVT can worsen hepatocellular damage and portal hypertension due to vascular occlusion and hepatocyte ischemia etc., ultimately resulting in liver failure and upper gastrointestinal bleeding, which could threaten the life and health of cirrhotic patients [2, 3]. Therefore, the prevention and treatment of PVT are important strategy for patients with liver cirrhosis.

The pathogenesis of PVT in liver cirrhosis may be multifactorial and complicated. Tripodi et al. ’s “Virchow triad” theory can be used to explain PVT [4], which includes slowed blood flow, blood hypercoagulation and vascular endothelial damage. However, there were lots of debates for this well-known complication [5], much more studies are needed to elucidate the underlying mechanisms of PVT development in cirrhosis. The treatment for PVT in cirrhosis is challenging, the current options include anticoagulation therapy, thrombolysis and transjugular intrahepatic portosystemic shunt (TIPs). Among them, rivaroxaban (RIVA), a direct-acting oral anticoagulant with highly selective inhibitory action against Factor Xa, was approved for clinical use in the prevention of venous thromboembolism in 2008, later applied for PVT widely and effectively [6]. However, the more ideal treatment of PVT in cirrhosis are still needed given the above-mentioned options have limitations or side effects.

At present, there is no suitable animal model of PVT caused by liver cirrhosis. Rabbit, dog, pig, baboon or other large animal models have high experimental costs and lack cirrhotic histology [7], which limits their application in investigation of pathological mechanisms of PVT and development of antithrombotic drugs. Studies had proven that homozygous mouse with the factor V Leiden (FVL) mutation can develop occlusive thrombosis more quickly than wild-type mice [8]. However, this model was impractical, because this mutant mouse not only was expensive but also needed to be self-reproduced and subsequently identified, and it was difficult to breed transgenic mice.

In view of the unmet needs for the mechanisms understanding and treatment development of PVT with cirrhosis, it is highly important to establish a practical animal model. It was well known that Carbon tetrachloride (CCl_4_)-induced liver fibrosis and cirrhosis are widely accepted experimental models that may induce micro thrombosis [9]. The ligation of main portal vein could cause the PVT in animal, which is characterized by thickening of the portal vein wall, swelling of endothelial cells and mural thrombosis [10]. Therefore, in this study, we integrated and modified the above methods, attempted to establish a rat model of liver cirrhosis complicated with PVT, by partial portal vein ligation (PPVL) plus CCl_4_ intoxication for simulating liver fibrosis and cirrhosis. Meanwhile, the models were treated with RIVA, and the features and mechanisms of PVT pathological formation and RIVA pharmacological action were explored, relating to blood flow at portal vein, blood coagulation, endothelial cell injury and thrombolysis.

## Materials and methods

### Drugs and main agents

Drugs: RIVA, an oral direct inhibitor of factor Xa, was purchased from ChemBest Research Laboratories Ltd. (CAS No. 366789-02-8).

Biochemical Reagents: alanine aminotransferase (ALT) kit (Cat No. C009-1) and aspartate aminotransferase (AST) kit (Cat No. C010-1) were purchased from Nanjing Jiancheng Institute of Biological Engineering. The hydroxyproline standard (trans-4-hydroxy-L-proline, Cat No. H54409) was purchased from Sigma.

Antibodies and pathological testing reagents: SABC Immunohistochemical Staining Kit (Cat No. SA1028-Rabbit IgG), anti-PAI-1 antibody (Cat No. ab66705), anti-fibrinogen rabbit monoclonal antibody (Cat No. ab189490), and anti-von Willebrand factor (vWF)antibody (Cat No. ab6994) were all purchased from Sigma.

### Establishment of a PVT model with cirrhosis in rats

**Fig. 1:**
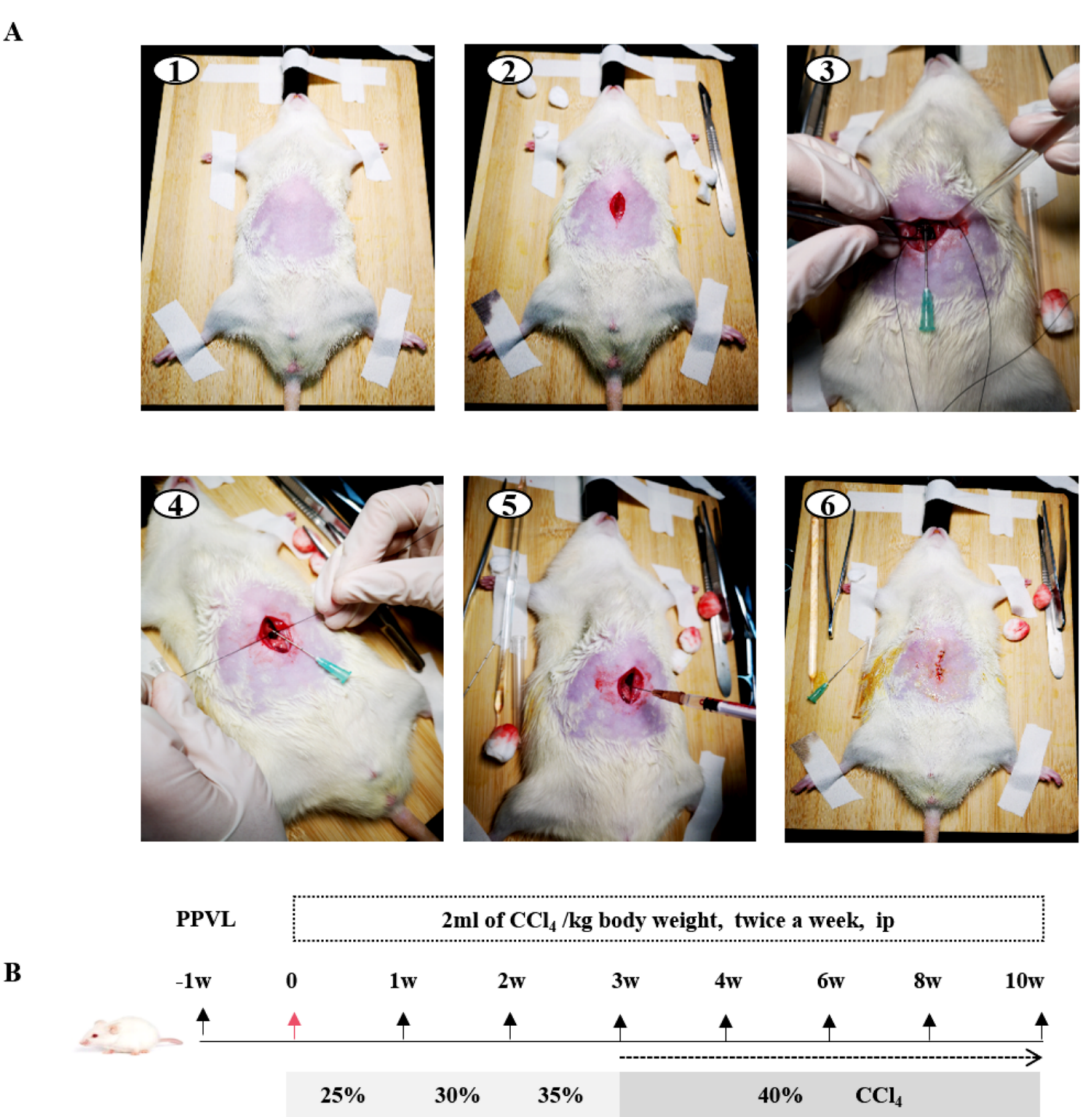
An animal model of portal vein thrombosis with cirrhosis in rats was established by partial portal vein ligation (PPVL) and CCl_4_ intoxication. **(A)** Surgical procedures for PPVL: (A1) Skin preparation and disinfection. (A2) A longitudinal incision approximately 1.5–2 cm was made along the midline of the abdomen. (A3) A 20G needle was placed adjacent to over the portal vein. (A4) The portal vein was ligated together with a 20G needle using silk thread, after which the needle was slowly withdrawn. (A5) Penicillin was injected. (A6) The abdomen and skin were closed. **(B)** Flowchart of model establishment. First, 160–180 g rats underwent the PPVL operation. One week later, the rats were injected intraperitoneally with 25-40% CCl_4_ at a volume of 2 ml/kg body weight twice a week and divided into different model groups according to the following time points: 4 weeks (*n* = 6), 6 weeks (*n* = 6), 8 weeks (*n* = 6), and 10 weeks (*n* = 10)

Fifty-six male Sprague‒Dawley rats weighing 160–180 g were obtained from Beijing Vital River Laboratory Animal Technology Co., Ltd. Among them, 7 rats were used as the normal control, other 49 rats received PPVL operation [11], one week later the survival rats were intraperitoneal injected with CCl_4_ for 4–10 weeks, as shown in the flow chart in Fig. 1B. Briefly, the rat skin was disinfected (Fig. 1-A1), 1.5–2 cm longitudinal incision was made in the upper abdomen (Fig. 1-A2). After the main portal vein was exposed and isolated, a 20G needle was placed adjacent to the portal vein, after which the portal vein was ligated with silk thread (Fig. 1-A3). At this time, the small intestine and stagnant mesenterial veins became stabile, and the color of these veins turned dark blue or violet (Fig. 1-A4). Then, the needle was removed slowly, which partially restored the portal vein blood flow. After penicillin was injected intraperitoneally (Fig. 1-A5), the peritoneum and the skin were closed separately (Fig. 1-A6). After one week, the rats that received PPVL were injected intraperitoneally with different doses of CCl_4_ for 10 weeks, as shown in Fig. 1B.

### Experimental design

The survival rats after one week of PPVL were randomly divided into 6 groups according to random number table: the PPVL control group (without CCl_4_ intoxication; *n* = 6), the 4 week model group (*n* = 6), the 6 week model group (*n* = 6), the 8 week model group (*n* = 6), the 10 week model group (*n* = 10) and the RIVA-treated group (*n* = 10). All rats in model groups were intraperitoneally injected with 25-40% CCl_4_ dissolved in olive oil twice a week for 4 weeks to 10 weeks, the rats in RIVA-treated group received 10 weeks of CCl_4_ intoxication and orally took RIVA (20 mg/kg body weight [9]) for 6 weeks from the 4th weekend to 10th weekend. While 7 rats with the same species and weight took the same volume of saline as normal control group (*n* = 7). All animal experiments were approved by the institutional animal ethics committees of the Laboratory Animal Center at Shanghai University of Traditional Chinese Medicine, Shanghai, China (Ethics Number: SZY201804011). The euthanasia of rats was performed with sodium pentobarbital intraperitoneally 150–200 mg/kg of rat weight, which were complied with our university guideline for animals ethnic.

### Biochemical tests

After opening the abdominal cavity, blood was collected from the abdominal aorta. The sera were collected from the rats and assayed for ALT and AST levels with kits. In addition, 4 ml of plasma was collected from each rat treated with anticoagulation agents, aliquoted into 2 tubes, and applied for the detection of platelet (PLT), Fibrinogen (FIB), D-Dimer and AT III by the Laboratory Center of Shuguang Hospital Affiliated with the Shanghai University of Traditional Chinese Medicine.

### Hepatic hyp content assay

The hydroxyproline (Hyp) content in the tissue was measured with Jamall’s method [12]. Briefly, 100 mg of liver sample was homogenized and hydrolyzed in 12 M HCl at 110 °C for 18 h. After filtration of the hydrolysate, chloramine T was added to a final concentration of 2.5 mM for 10 min at room temperature. The mixture was then treated with 25% (w/v) p-dimethylaminobenzaldehyde and 27.3% (v/v) perchloric acid in isopropanol and incubated at 50 °C for 90 min. At last, the samples were examined at 558 nm against a reagent blank. The concentration of Hyp in each sample was determined from a standard curve, which was generated from a series of known quantities of Hyp ranging from 0.2 to 1.6 µg (Peptide Co., Japan). The Hyp concentration is expressed as µg/g of liver weight.

### Histological examinations

Collected liver tissues were fixed in 4% formalin and embedded in paraffin. Sections (4 μm) were stained with hematoxylin-eosin (HE) and Sirius red.

### Immunohistochemistry

Paraffin-embedded slices (4 μm) were subjected to immunohistochemical staining. Endogenous peroxidase activity was blocked by methanol with 3% H_2_O_2_ and bovine serum albumin (BSA). After washing with PBS, the sections were incubated with the following primary antibodies at 4 °C overnight: anti-fibrinogen antibody (ab189490, 1:1500) and anti-PAI1 antibody (ab66705, 1:200). On the second day, the sections were incubated with horseradish peroxidase (HRP)-conjugated secondary antibody for 1 h at 37 °C. Diaminobenzidine (DAB) was used as a chromogen, followed by hematoxylin counterstaining.

### Immunofluorescence

The collected liver tissues were put into Tissue-Tek OCT embedding medium and snap-frozen in liquid nitrogen. Then, the tissues were fixed with acetone for 10 min, washed with PBS, and blocked with 0.5% BSA for 1 h at 37 °C. The tissues were incubated with the primary antibody Anti-vWF (ab6994, 1:200) at 4 °C overnight. The next day, fluorescein isothiocyanate-labeled secondary antibodies were added to the samples, which were incubated for 1 h at 37 °C. Subsequently, DAPI (ab228549, 1:1000) was used to stain the nucleus. The tissues were observed under a confocal microscope for imaging.

### Western blot analysis

Liver lysates were separated on 10% SDS‒PAGE gels and transferred to nitrocellulose membranes. The membranes were blocked with 5% BSA and incubated with different primary antibodies: anti-fibrinogen antibody (ab92572, 1:1000) or anti-PAI-1 antibody (ab66705, 1:1000) at 4 °C overnight. After three washes with PBS, the membranes were incubated with secondary antibodies at room temperature for 1 h. After an additional three washes, the membranes were scanned and imaged with Li-Cor odyssey.

### Portal vein ultrasonic detection

An experienced B-mode ultrasound doctor applied Doppler ultrasound (Philips IU22) to observe portal vein thrombosis, portal vein blood flow velocity, and portal vein diameter in rats, with the special probe for animal. The ultrasound frequency was set to 11 M, and the blood flow angle was < 60°.

### Statistical analysis

All the data were analyzed by using SPSS software version 26.0. Differences between the groups were assessed by nonparametric one-way analysis of variance (ANOVA) followed by the least significant difference (LSD) post hoc test. Quantitative data are expressed as the mean ± standard deviation (SD). A *P* value < 0.05 was considered to indicate statistical significance.

## Results

### The characteristics of the PVT model in liver cirrhotic rats

**Fig. 2:**
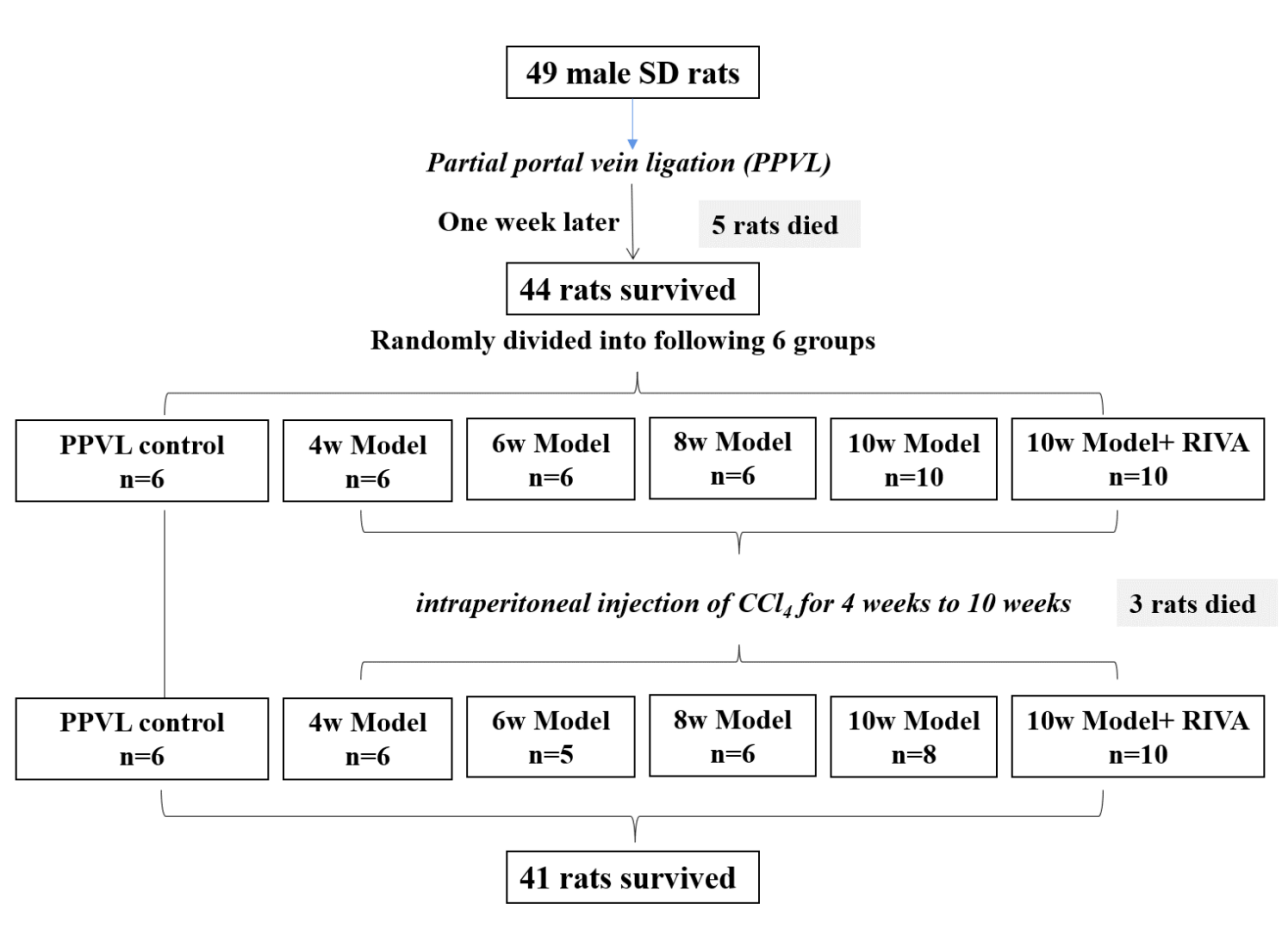
The group distribution of model rats and the numbers of deaths and survivals

49 rats received PPVL, one week later, 44 survived rats were divided into different groups and intoxicated with CCl_4_ for 4–10 weeks except PPVL control group. At last, 41 model rats survived, with total survival rate of 83.7%, the death reasons of model rats may include the abdominal infection, surgical injury and CCl_4_ intoxication. The group distribution of the model rats as well as the numbers of deaths and survivals were shown as Fig. 2.

**Fig. 3:**
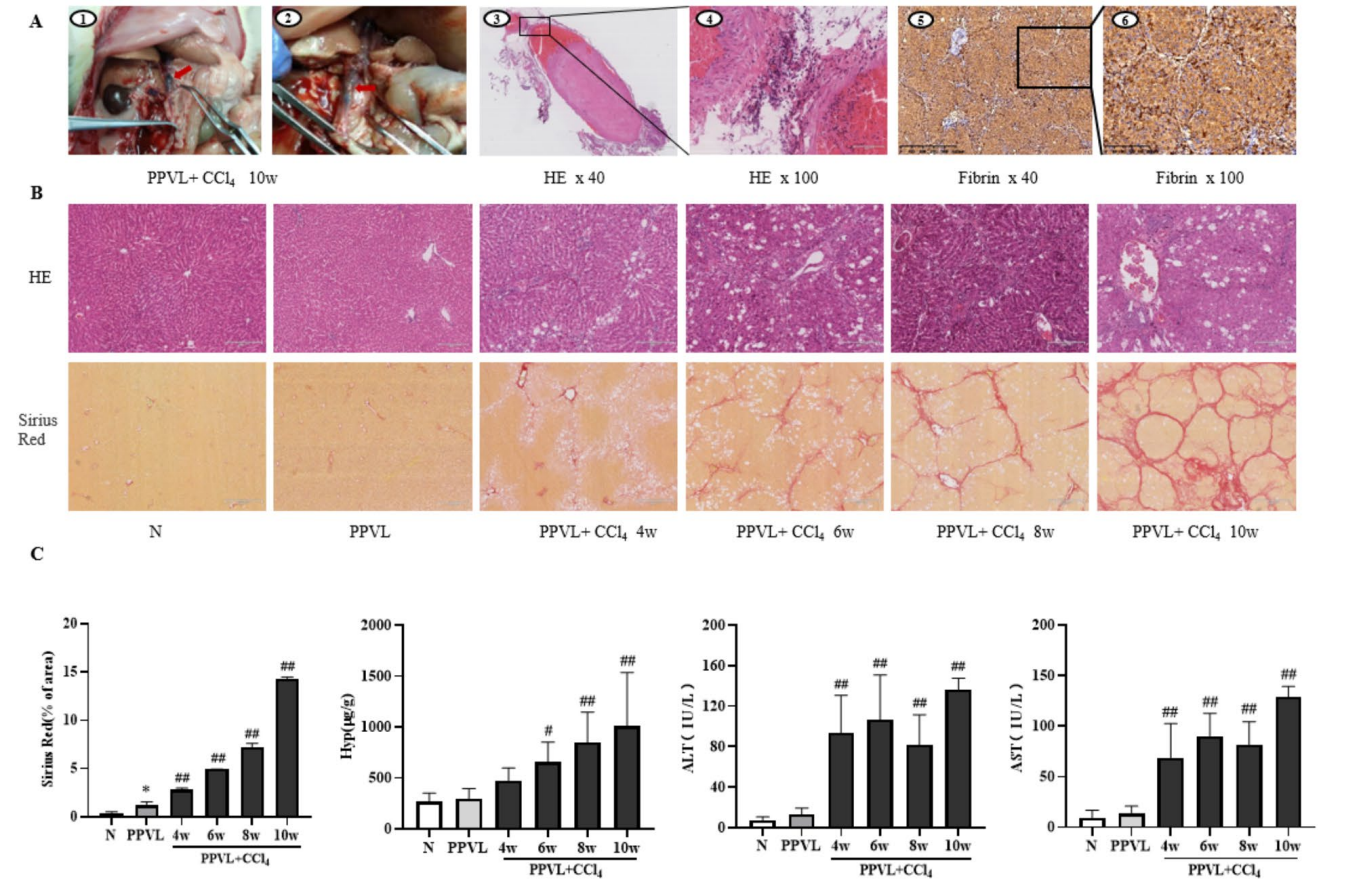
The establishment of a PVT model in rats with liver cirrhosis and characteristics of extrahepatic macrothrombi and intrahepatic microthrombi. **(A)** Gross appearance of portal vein thrombosis. As indicated by the arrow, a segment approximately 0.5 cm in length protruded with the black thrombi at the site of ligation of the portal vein (A1, A2). Portal vein HE staining (A3, ×40; A4, ×100). Fibrin immunohistochemical staining (A5, ×40; A6, ×100). **(B)** Representative images of H&E-stained liver sections (upper panels, ×200) and Sirius red staining (lower panels, ×100) of rat liver tissues at different model groups. **(C)** The semi-quantitation of Sirius red staining (collagen deposition) in liver, hepatic hydroxyproline content, serum ALT and AST activities of different model groups. The data are expressed as the mean ± SD. N (*n* = 7); PPVL (*n* = 6); PPVL + CCl_4_ 4w (*n* = 6); PPVL + CCl_4_ 6w (*n* = 5); PPVL + CCl_4_ 8w (*n* = 6); PPVL + CCl_4_ 10w (*n* = 8); ^*^P < 0.05 vs. N, ^#^*P* < 0.05 vs. PPVL, ^##^*P* < 0.01 vs. PPVL

After the PPVL and 10 weeks of CCl_4_ intoxication, the rats had obvious thrombi in portal vein and stenosis outside the liver (Fig. 3-A1 and A2). HE staining revealed that the portal vein comprised thrombi accompanied by the infiltration of many inflammatory cells. The vascular wall exhibited thickening, roughening of the intima, severe damage, and even exfoliation, with noticeable swelling of the endothelial cells (Fig. 3-A3 and A4). Immunohistochemical staining confirmed that fibrin, an important component of microthrombi, was abundantly expressed in the sinusoids and a few in hepatocyte in the liver of model rat, and remarkably at 10 weeks models, the typical slides were shown as Fig. 3-A5 and A6, and dynamic change showed in Fig. 5B. These results indicated that the rat model had both macro thrombi in the portal veins as well as micro thrombi in the liver.

In the model rats, the serum ALT and AST levels increased significantly after 4 weeks of CCl_4_ administration compared to the normal group. HE and Sirius red staining indicated that the model rats had hepatic inflammation, necrosis and liver fibrosis at 6th week and cirrhosis at 10th week. The hepatic Hyp also gradually increased as the CCl_4_ intoxication prolonged. These results confirmed that the model rats had a progression of liver fibrosis during modeling and developed into cirrhosis at 10th week.

### Portal vein blood flow velocity decreased in the rat model of cirrhotic PVT

**Fig. 4:**
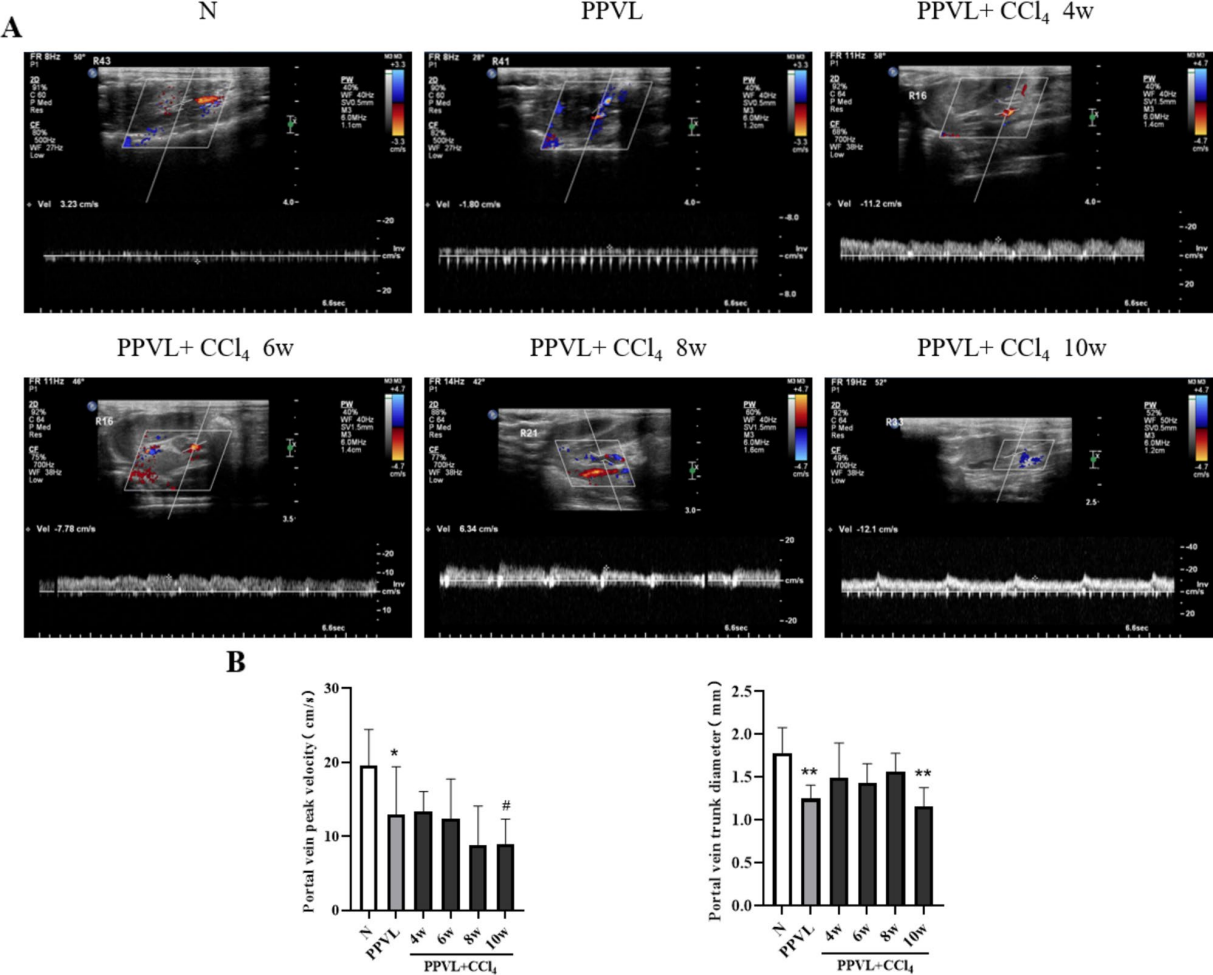
The blood flow velocity and trunk diameter at portal vein in the PVT model in rats with liver cirrhosis. **(A)** Portal vein ultra- sonographies at different rat model groups. **(B)** Peak velocity and trunk diameters of the portal vein at different rat model groups. The data are expressed as the mean ± SD. N (*n* = 7); PPVL (*n* = 6); PPVL + CCl_4_ 4w (*n* = 6); PPVL + CCl_4_ 6w (*n* = 5); PPVL + CCl_4_ 8w (*n* = 6); PPVL + CCl_4_ 10w (*n* = 8); ^*^P < 0.05 vs. N, ^**^P < 0.01 vs. N, ^#^*P* < 0.05 vs. PPVL

After modeling, the diameter of rat portal vein trunk became obviously narrower than that in the normal rats due to PPVL, and portal blood flow velocity (PBFV) was also slower in the PPVL control group than one in the normal group (*P* < 0.05), as shown in Fig. 4. Among the model rats, the blood flow in the PPVL + CCl_4_ 10w group was significantly slower than that in the PPVL control group (*P* < 0.05) (Fig. 4B).

### Pro- and anti- coagulation functions were decreased, but relative hypercoagulable state vulnerable to be embolized in cirrhotic PVT in rats

Continued CCl_4_ administration after PPVL deteriorated the coagulation functions of the model rats, as shown in Table 1. The pro-coagulant factor, such as fibrinogen (FIB), was decreased in the serum of model rats. Meanwhile, anticoagulant factors, such as antithrombin III (AT III), were also decreased, which indicated that model rats stated at a lower level of pro-coagulable and anti-coagulable functions. While FIB/AT III ratio increased in the model rats, particularly in the 10-week model rats. This indicated that the model rats had relative hypercoagulable state, which was supported by an increase of serum D-Dimer, and was prone to clot formation in the model rats.

**Table 1:** Dynamic changes of blood coagulant factors in model rats (mean ± SD)

Group	*n*	FIB(×10^3^mg/L)	D-Dimer (ug/ml)	AT III (%)	PLT(×10^9^/L)	FIB/AT III
N	7	3.97 ± 1.04	0.21 ± 0.07	119.71 ± 7.02	941.14 ± 190.91	33.01 ± 7.81
PPVL	6	3.45 ± 1.21	0.17 ± 0.04	118.83 ± 6.40	752.67 ± 96.79	28.68 ± 8.34
4 WM	6	2.87 ± 0.32	0.25 ± 0.07	98.67 ± 10.82	886.33 ± 232.80	29.26 ± 3.19
6 WM	5	2.98 ± 0.66	0.36 ± 0.26	85.60 ± 23.85	596.20 ± 223.85	35.63 ± 5.71
8 WM	6	3.17 ± 0.69	0.20 ± 0.06	109.67 ± 10.11	780.83 ± 166.13	28.78 ± 5.45
10 WM	8	2.42 ± 0.40^#^	0.48 ± 0.29^#^	67.63 ± 15.16^##^	806.13 ± 262.55	36.53 ± 4.91^#^

N: control group; PPVL: partial portal vein ligation group; 4 WM: 4-week model group; 6 WM: 6 week model group; 8 WM: 8 week model group; 10 WM: 10 week model group

^*^P < 0.05 vs. N, ^**^P < 0.01 vs. N; #*P* < 0.05 vs. PPVL, ##*P* < 0.01 vs. PPVL

### Vascular endothelial injury and expression of fibrin and PAI-1 in PVT model rats

**Fig. 5:**
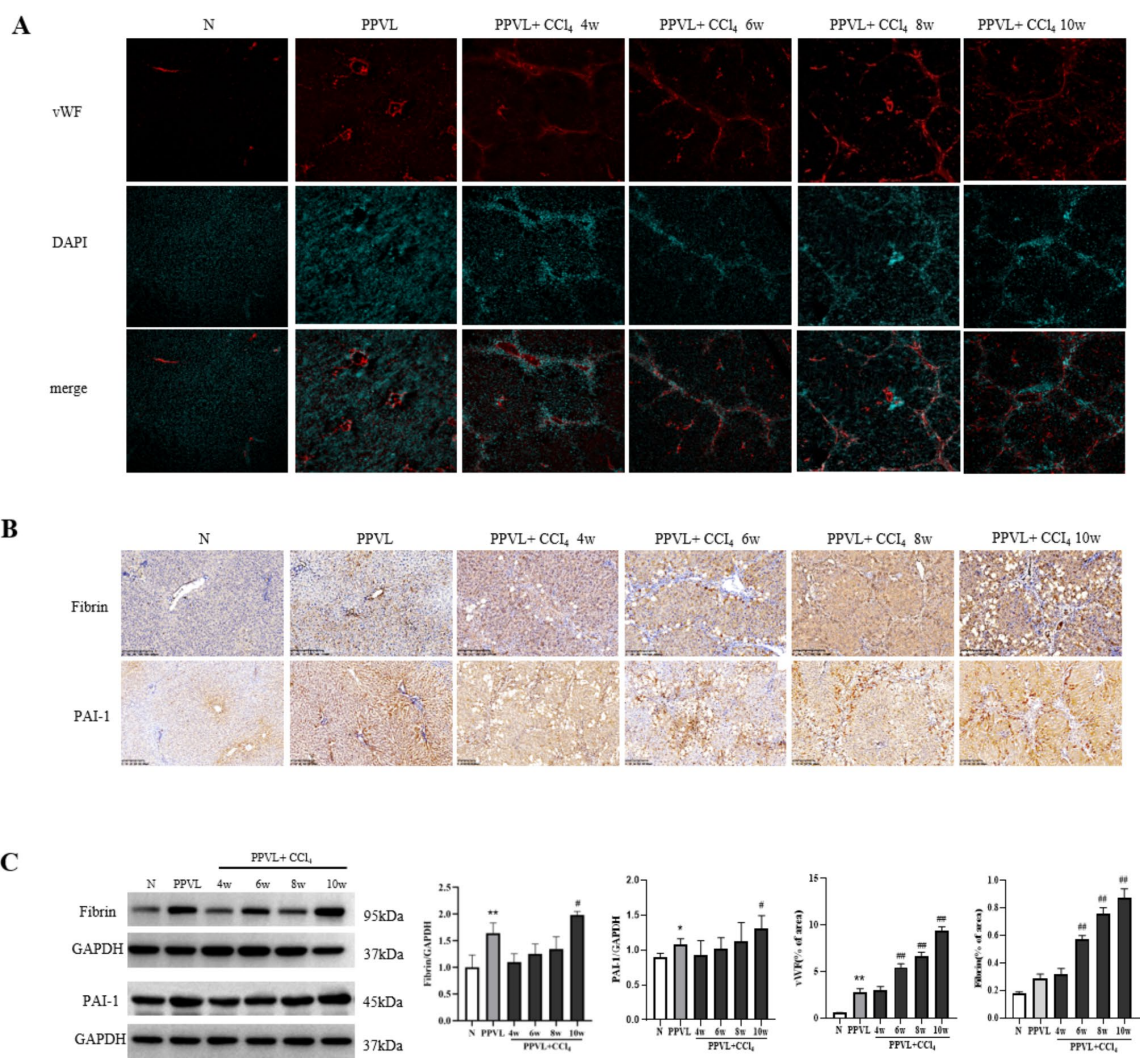
Vascular endothelial injury and fibrin deposition in the PVT model rats. **(A)** Immunofluorescence staining of vWF in rat liver tissue (×100). **(B)** Immunohistochemical staining of Fibrin and PAI-1 (×100). **(C)** C1. Western blot showing Fibrin and PAI-1 protein levels and semiquantitative analysis by Image J (C1, C2, C3). Semiquantitative analysis of vWF (C4). Semiquantitative analysis of Fibrin (C5). The data are expressed as the mean ± SD; *n* = 3. *P < 0.05 vs. N, **P < 0.01 vs. N, ^#^*P* < 0.05 vs. PPVL, ^##^*P* < 0.01 vs. PPVL

vWF, a marker of vascular endothelial injury, was increased gradually at 6-weeks, 8-week and 10-week models compared with those in the PPVL control group (*P* < 0.01), as depicted in Fig. 5A. Fibrin was converted from fibrinogen via thrombin, mainly located in sinusoids and partly in hepatocytes, and formed the clots or microthrombi in diseased liver. As the CCl_4_ intoxication continued, the model rats had gradually increased expressions of hepatic fibrin, demonstrated by immunohistochemical staining and western blot analysis (Fig. 5-B and C3). Moreover, the model rats had increased expression of PAI-1, which is an endogenous inhibitor of fibrinolysis, in particular at 10-week model. (Fig. 5-B and C). It suggested that the model rats had an increase of intrahepatic micro thrombi as liver fibrosis progressed, with impaired thrombolytic function.

### Rivaroxaban reduces thrombosis and liver fibrosis in model rats

**Fig. 6:**
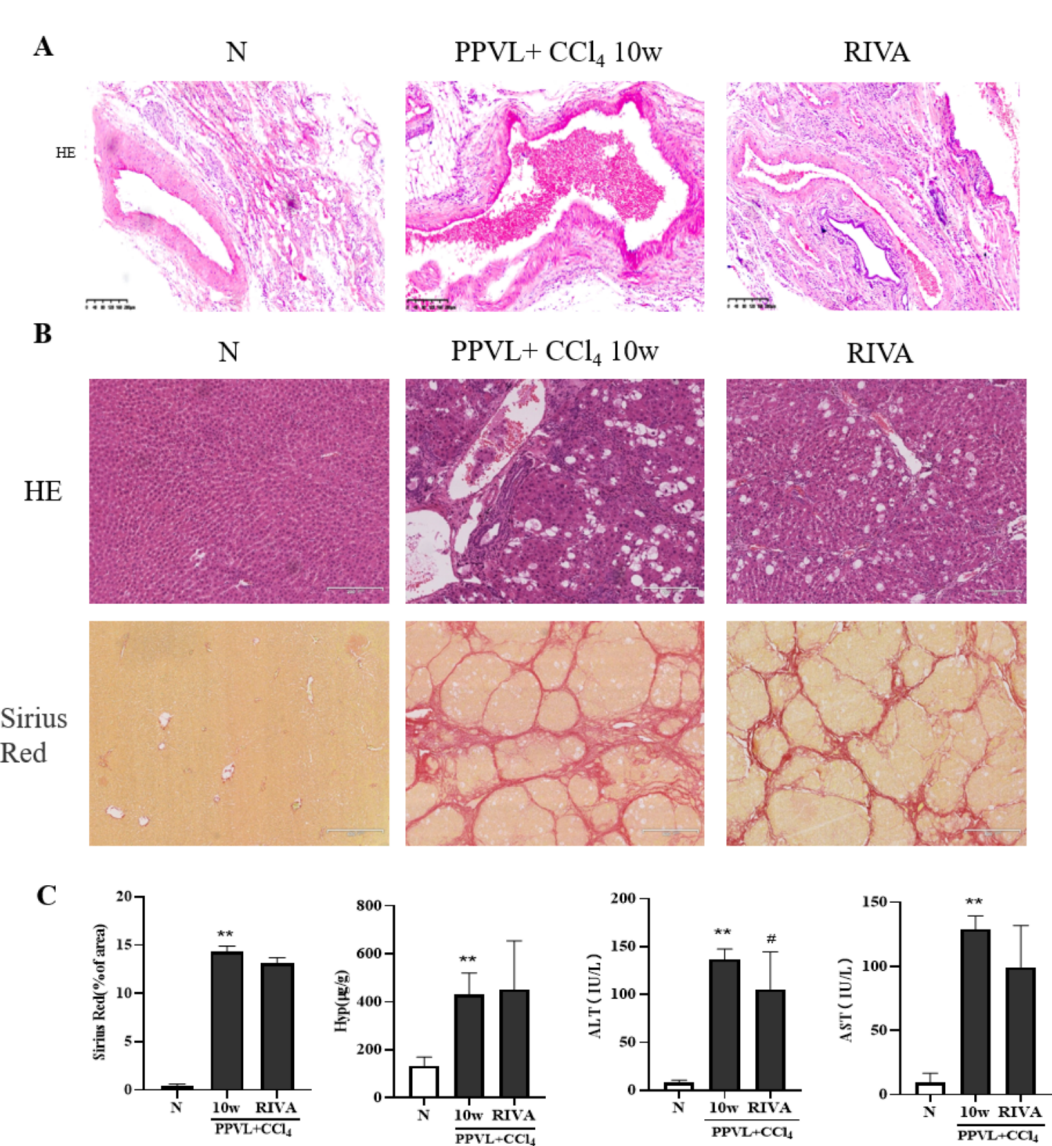
Rivaroxaban reduces thrombosis and attenuates liver inflammation in model rats. **(A)** HE staining (×100) of rat portal veins after RIVA treatment. **(B)** HE staining (upper panels, ×200) and Sirius red staining (lower panels, ×100) of rat liver tissues after RIVA treatment. **(C)** Effects of RIVA on the serum ALT, AST and hepatic Hyp contents of the model rats. The data are expressed as the mean ± SD; ^*^P < 0.05 vs. N, ^**^P < 0.01 vs. N, ^#^*P* < 0.05 vs. PPVL + CCl_4_ 10w, ^##^*P* < 0.01 vs. PPVL + CCl_4_ 10w

We treated the model rats with RIVA for 6 weeks from 4th weekend to 10th weekend, and found that the drug could decrease portal vein thrombi, as shown in Fig. 6A. Moreover, the drug attenuated the serum ALT and AST levels and decreased liver inflammation, as shown by HE staining. However, there was no significant difference between PPVL + CCl_4_ 10w model and RIVA treated group for the hepatic Hyp content and collagen deposition by Sirius red staining, as shown in Fig. 6. These results suggested that RIVA improved portal vein thrombosis but had no obvious effect on liver fibrosis.

### Rivarosaban prevents hypercoagulable state and increases PBFV in model rats

RIVA improved the coagulation function of the model rats, as shown in Table 2. Compared with PPVL + CCl_4_ 10w model rats, the RIVA-treated rats increased AT III significantly (*P* < 0.01), tend to increase FIB, but decreased FIB/AT III ratio significantly (*P* < 0.01).

**Table 2:** Effect of RIVA on the coagulant factors of model rats (mean ± SD)

Group	*n*	FIB(×10^3^mg/L)	D-Dimer (ug/ml)	AT III (%)	PLT(×10^9^/L)	FIB/AT III
N	7	3.97 ± 1.04	0.21 ± 0.07	119.71 ± 7.02	941.14 ± 190.91	33.01 ± 7.81
10 WM	8	2.42 ± 0.40**	0.48 ± 0.29*	67.63 ± 15.16**	806.13 ± 262.55	36.53 ± 4.91
RIVA	10	2.83 ± 0.35	0.32 ± 0.34	100.60 ± 13.88^##^	899.90 ± 237.85	28.41 ± 3.64^##^

N: control group; 10 WM: PPVL + CCl_4_ 10w group; RIVA: RIVA-treated group. ^*^P < 0.05 vs. N,

^**^P < 0.01 vs. N; ^#^*P* < 0.05 vs. 10 WM, ^##^*P* < 0.01 vs. 10 WM

**Fig. 7:**
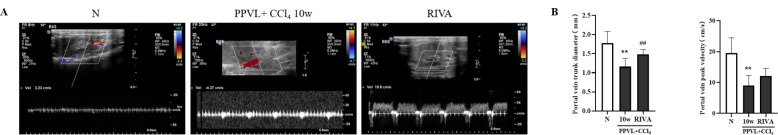
RIVA improved the portal vein diameter and blood flow velocity in model rats. **(A)** Portal vein ultrasonography after RIVA treatment. **(B)** Comparison of the blood flow velocity through the portal vein in each group. The data are expressed as the mean ± SD; N, *n* = 7; PPVL + CCl_4_ 10 weeks, *n* = 8; and RIVA, *n* = 10. ^*^P < 0.05 vs. N, ^**^P < 0.01 vs. N, ^#^*P* < 0.05 vs. PPVL + CCl_4_ 10w, ^##^*P* < 0.01 vs. PPVL + CCl_4_ 10w

Ultrasonography revealed that the portal vein diameter was significantly wider in the RIVA-treated rats than in PPVL + CCl_4_ 10w model rats (*P* < 0.01), and the blood flow velocity tended to increase; however, there was no statistically significant difference, as shown in Fig. 7-A and B.

### Rivaroxaban alleviates vascular injury and promotes fibrin degradation in model rats

**Fig. 8:**
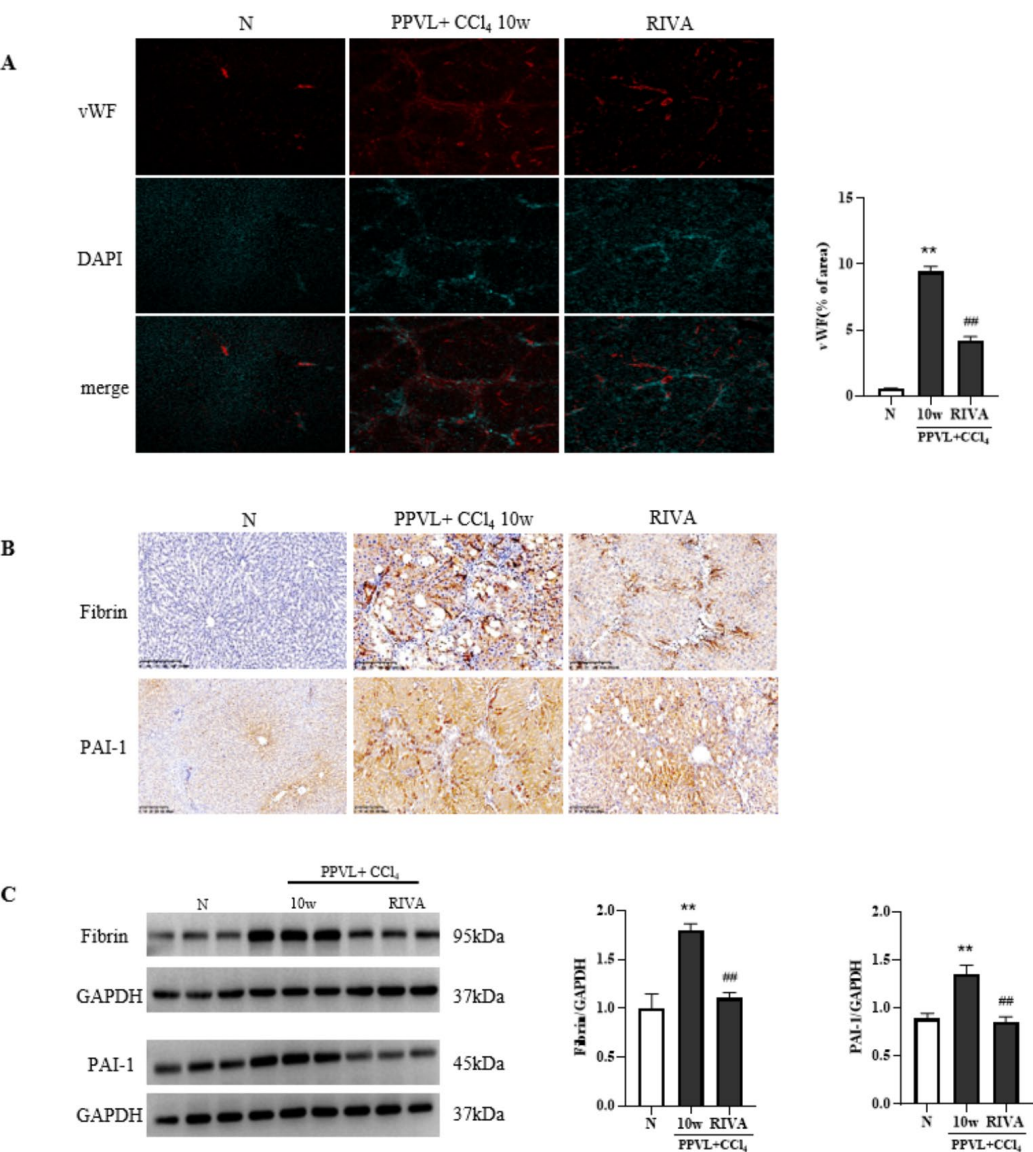
Rivaroxaban alleviates vascular injury in model rats and promotes fibrin degradation. **(A)** Immunofluorescence analysis of vWF after RIVA treatment (×100). **(B)** Immunohistochemical staining of Fibrin and PAI-1 after RIVA treatment (×100). **(C)** Western blot showing Fibrin and PAI-1 protein levels and semiquantitative analysis by ImageJ. The data are expressed as the mean ± SD; *n* = 3. ^*^P < 0.05 vs. N, ^**^P < 0.01 vs. N, ^#^*P* < 0.05 vs. PPVL + CCl_4_ 10w group, ^##^*P* < 0.01 vs. PPVL + CCl_4_ 10w group

RIVA-treated rats had significant decrease in vWF expression than PPVL + CCl_4_ 10w group (*P* < 0.01), as shown in Fig. 8A, indicating that RIVA might improve the injury in vascular endothelial cell. Additionally, the expression of fibrin and PAI-1 in RIVA-treated rats was lower than that in the PPVL + CCl_4_ 10w group, as demonstrated by immunohistochemical staining and western blotting (Fig. 8-B and C). This finding indicates that RIVA may promotes thrombolysis and fibrin degradation.

## Discussion

PVT had a close relationship with liver cirrhosis, its incidence remarkably increased as liver function and portal hypertension in cirrhotic patients deteriorated, also PVT development may worsen the outcome of patients with cirrhosis [13]. A systematic review indicates that PVT raises the risk of variceal bleeding [3], rebleeding in cirrhotic patients, and is associated with high risks of intractable ascites and hepatorenal syndrome, thereby impacting patient prognosis. Therefore, the management of liver cirrhosis complicated by PVT assumes paramount importance. Despite existing the clinical consensus for PVT with cirrhosis, there were numerous unmet needs for clinic practice [14, 15]. For example, the anticoagulant was recommended treatment for the cirrhotic PVT, but recently Driever EG et al. found that 2/3 of patients with nonmalignant portal vein thrombi were refractory to anti- coagulation agents [16]. Thus, there exists an urgent imperative to establish animal models that faithfully replicate the clinical characteristics of liver cirrhosis complicated by PVT, in order to facilitate the investigations into the pathophysiological mechanisms and the developing the novel medicinal for the PVT within liver cirrhosis.

However, the animal model of PVT caused by liver cirrhosis was still not available yet. Although the partial portal vein ligation could induce the intra and extra portal vein thrombosis in rats [14], this acute PVT model lacks the background of chronic liver damage including liver fibrosis, and tends to recover automatically. In our study, we at first partially ligated the portal vein trunk, then induced liver inflammation and fibrosis through intraperitoneal injection of varying doses of CCl_4_ for 10 weeks. As a result, the rats exhibited both obvious characteristics of portal vein thrombosis and liver cirrhosis. The thrombosis in the rat model had not only occurred at portal veins with macro thrombi which was visible to naked eyes and confirmed by HE staining, but happened widely in hepatic sinusoids with remarkable microthrombi evidenced by fibrin deposition. Fibrin was converted from fibrinogen via the action of thrombin, mainly located in sinusoids, and formed the clots with fibronectin and blood cells in cirrhotic liver, and can reflect the micro thrombosis in liver [17]. Subsequent CCl_4_ exposure gradually induced liver inflammation and fibrosis, and developed into cirrhosis confirmed by the fibrotic architecture in liver tissue and increased hepatic Hyp contents. This fibrogenic lesions also contributed to PVT development by reducing portal vein blood flow and disrupting the coagulation function etc. As far as we know, this is the first time the PVT model was modified through PPVL plus CCl_4_ intoxication.

The relation between the PVT and liver cirrhosis and the mechanisms underlying PVT pathogenesis in cirrhosis are not fully elucidated, given the very complicated circumstances for PVT or cirrhosis itself and interaction between two events. However, the “Virchow triad” theory was widely recognized for the risk factors of PVT development in cirrhosis, in which Tripodi et al. suggest that portal vein thrombosis mainly contributed to slowed blood flow, blood hyper-coagulation and vascular endothelial damage [4]. Although Zocco et al. reported that a decrease in portal vein blood flow velocity was the main predictor of cirrhosis [18], but the Stine JG et al. reported the portal vein velocity played an important role in PVT development in cirrhosis [19], when portal flow velocity falls below 15 cm/s, PVT risk significantly increases (*P* < 0.001). Our study also revealed that the blood velocity at the portal vein significantly decreased in the rat model of PVT in cirrhosis compared with the normal rats.

The liver can synthesize a lot of factors for coagulant function and contribute to health hemostasis. Recent year had witnessed the remarkable progress of understanding of cirrhotic coagulopathy [1]. The patients with advance liver disease had their coagulation system altered, both of pro- and anti- coagulant factors were decreased, but could rebalance to a new hemostatic system at much lower level. However, this rebalance was fragile and may tilt toward bleeding or thrombosis with minimal disturbance [20]. In the study, both the pro-coagulant factors (FIB etc.) and anti-coagulant factors (AT III, etc.) were decreased in the model rats, but the ratio of FIB to ATIII and D-dimer increased significantly in the 10-week model group compared with the normal and PPVL control, indicating that the model rats had relatively stronger pro-coagulant ability than anti-coagulant one, with a blood hypercoagulation state which was vulnerable to thrombosis in the diseased liver.

The vascular endothelium, including liver sinusoidal endothelial cells (LSECs), is main cellular basis of PVT. vWF could serve as markers for endothelial dysfunction [21]. Our study found that vWF was mainly located in sinusoidal area and increased in the model rats, indicating that vascular endothelial cell was injured in the model rats. While the enhanced vWF contributed to form thrombi as a component, and stimulated platelet activation to thrombosis. The endothelium plays a role not only in PVT formation but also degradation. Thrombolysis, a therapeutic option for PVT, relies on the fibrinolysis system. Vascular endothelial cells generate tissue-type plasminogen activator (t-PA), which converts plasminogen into plasmin, while plasmin could degrade the clots including fibrin into soluble fibrin degradation products (FDP) and promote thrombi resolution. The endothelial cells and hepatic stellate cell (HSC) in liver could produce PAI-1 [22, 23], which inactivates t-PA, inhibiting plasmin activity and impeding the thrombi degradation. While HSC activation produced extracellular matrix including collagens and contributed to liver fibrosis [24]. Our study did not fucus on HSC activation with α-SMA staining, but PAI-1 and collagen were increased in liver and sinusoids. The increased fibrin and PAI-1 levels in model rats indicated the down-regulated thrombolysis via plasmin, due to endothelial cell injury and HSC activation.

In the study, the RIVA was applied to treat 10-week model rats, the results showed that RIVA could efficiently reduce both visible thrombi at portal veins with HE staining and micro thrombi in liver mainly at hepatic sinusoidal area through fibrin staining, also improved the diameter and blood flow of portal veins in the model rats, indicated that RIVA had effects against PVT in cirrhosis in the model rat induced by PPVL plus CCl_4_ intoxication. Our results reconfirmed the effectiveness of RIVA on PVT in cirrhosis, and provide further justification for the reliability and practical significance of the rat model in the study. In addition, RIVA decreased vWF and PAI-1 in diseased liver of model rats, suggesting its action mechanism was associated with the reduction of thrombosis formation via ameliorating vascular endothelial cell damage, and the promotion of the clot degradation via PAI-1/plasmin pathway. RIVA, a potent and selective direct factor Xa inhibitor, exerts anticoagulant activity, and is ordinarily used for the patients with PVT in cirrhosis. In Vilaseca paper [9], it was found that RIVA decreases portal pressure in CCl_4_ and TAA induced rat models of cirrhosis, the effect is mostly associated to decreasing intrahepatic vascular resistance, reducing intrahepatic microthrombi, inhibiting HSC activation, enhancing NO bioavailability and improving endothelial cell dysfunction. Our findings added new understanding about RIVA effect on macro thrombi at portal veins, and the action mechanism with clot degradation.

Of course, there were limitations in our current study. For example, as a complication, PVT usually followed the cirrhosis of liver, our animal model was induced by physical blockage of blood flow and CCl_4_ intoxication, not fully mimic the natural history of cirrhotic PVT in patients. And the more in-depth molecular mechanism also needs to be explored in our future study. However, our current study provides a new animal model of PVT in cirrhosis, which could be helpful for further investigating the pathological mechanisms and searching for the novel treatments for PVT with cirrhosis.

In summary, we established a rat model of PVT with cirrhosis through PPVL plus CCl_4_ intoxication, and demonstrated that the model rats had characteristics of macrothrombi at portal veins and microthrombi in hepatic sinusoids, as well as liver cirrhosis. RIVA could attenuate PVT in cirrhosis model. The underlying mechanisms of PVT formation in the rat model and pharmacological action of RIVA are related to the regulation of portal blood flow, coagulant factors, and vascular endothelial cell function.

### Electronic supplementary material

Below is the link to the electronic supplementary material.

Supplementary Material 1

## Data Availability

The datasets generated and/or analyzed during the current study are available from the corresponding author or from: https://pan.baidu.com/s/1aXVdjKU--We6hVqrltUbaw?pwd=z980.

## Abbreviations

ALT: Alanine aminotransferase
AST: Aspartate aminotransferase
AT III: Antithrombin III
CCl_4_: Carbon tetrachloride
CDFI: Color Doppler flow imaging
FIB: Fibrinogen
FVL: Factor V Leiden
HE: Hematoxylin-eosin
HRP: Horseradish peroxidase
HSC: Hepatic stellate cell
Hyp: Hydroxyproline
LSECs: Liver sinusoidal endothelial cells
PAI-1: Plasminogen activator inhibitor − 1
PBFV: Portal blood flow velocity
PPVL: Part portal vein ligation
PLT: Platelet
PVT: Portal vein thrombosis
RIVA: Rivaroxaban
TIPs: Transjugular intrahepatic portosystemic shunt
t-PA: tissue-type plasminogen activator
vWF: Von Willebrand Factor
