# Supplementary material for: A new model of portal vein thrombosis in rats with cirrhosis induced by partial portal vein ligation plus carbon tetrachloride and intervened with rivaroxaban
Source: BMC Gastroenterol. 2024 May 13;24:161. doi: 10.1186/s12876-024-03253-4 (PMC11092107; doi:10.1186/s12876-024-03253-4)
Supplement: Supplementary file 1 — Supplementary Material 1 [file 12876_2024_3253_MOESM1_ESM.docx]

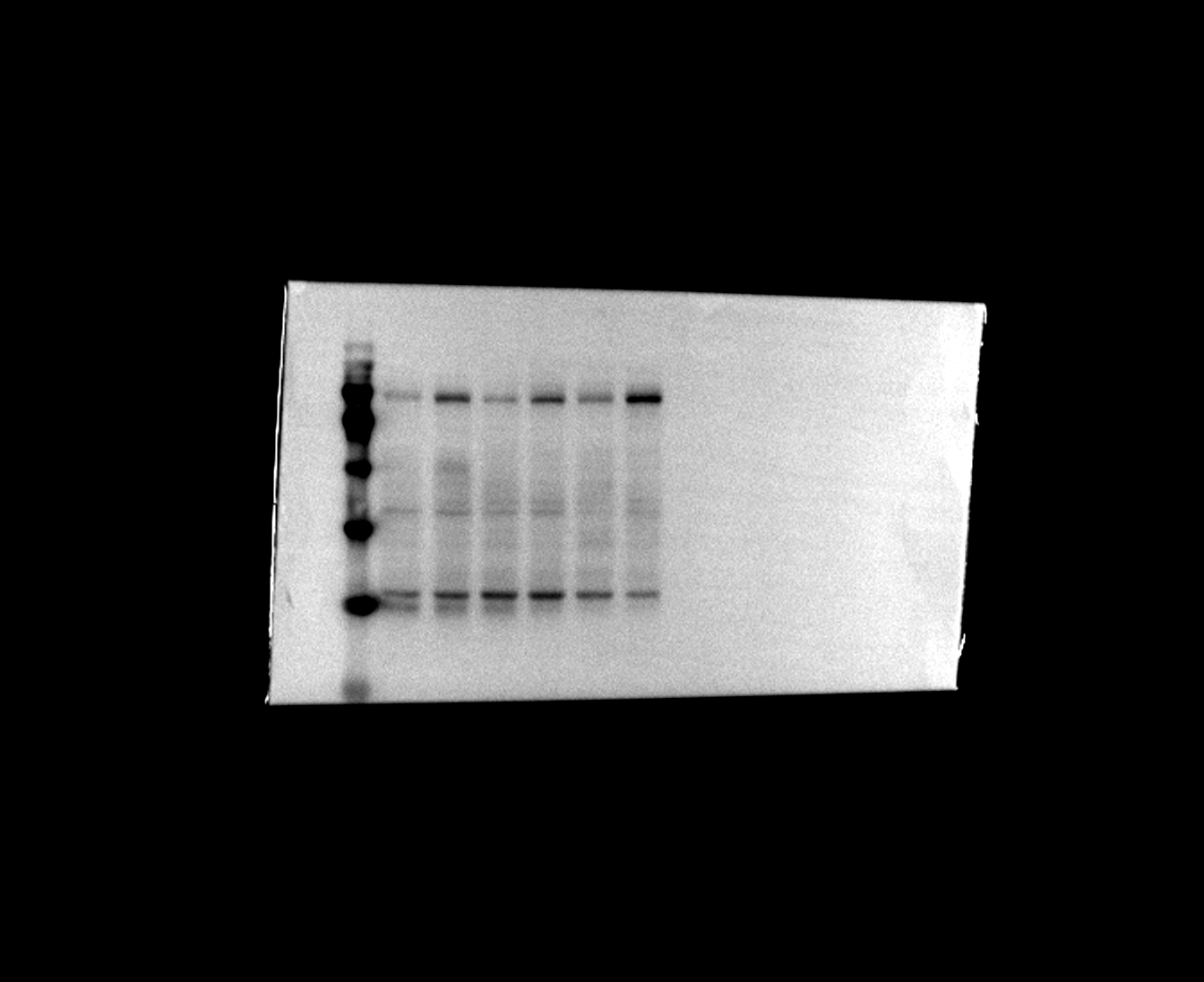


**95kD**

①Fibrin

**N PPVL 4W 6W 8W 10W**





①GAPDH

**N PPVL 4W 6W 8W 10W**

**37kD**





②Fibrin

**N PPVL 4W 6W 8W 10W**

**N PPVL 4W 6W 8W 10W**

**95kD**

**The representative bands forFibrin and GAPDH shown in Figure 5-C.**


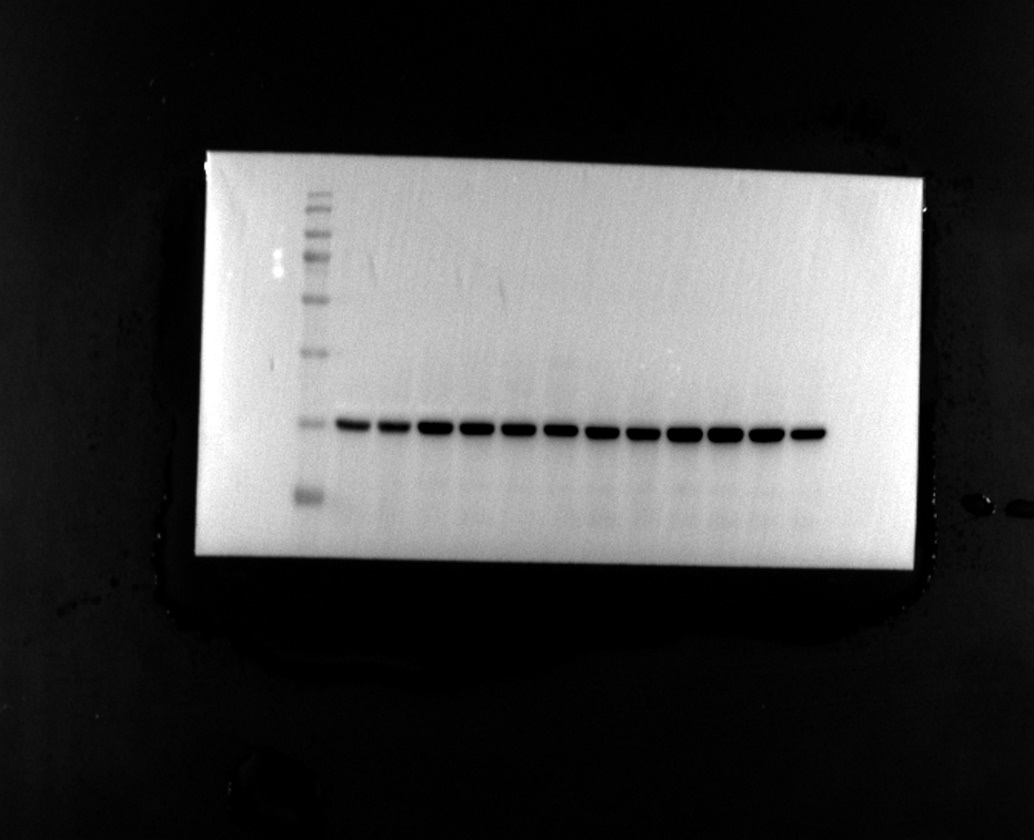


②GAPDH

**37kD**

**N PPVL 4W 6W 8W 10W**

**N PPVL 4W 6W 8W 10W**





**①**PAI-1

**45kD**

**N PPVL 4W 6W 8W 10W**

**The representative bands for PAI-1 and GAPDH shown in Figure 5-C**





**①**GAPDH

**N PPVL 4W 6W 8W 10W**

**37kD**

The second repeated group in right adjacent gels were also arranged with the order of “N PPVL 4W 6W 8W 10W”, but 10w band was missed at the second groups given the gels were not completely transferred to the membranes.


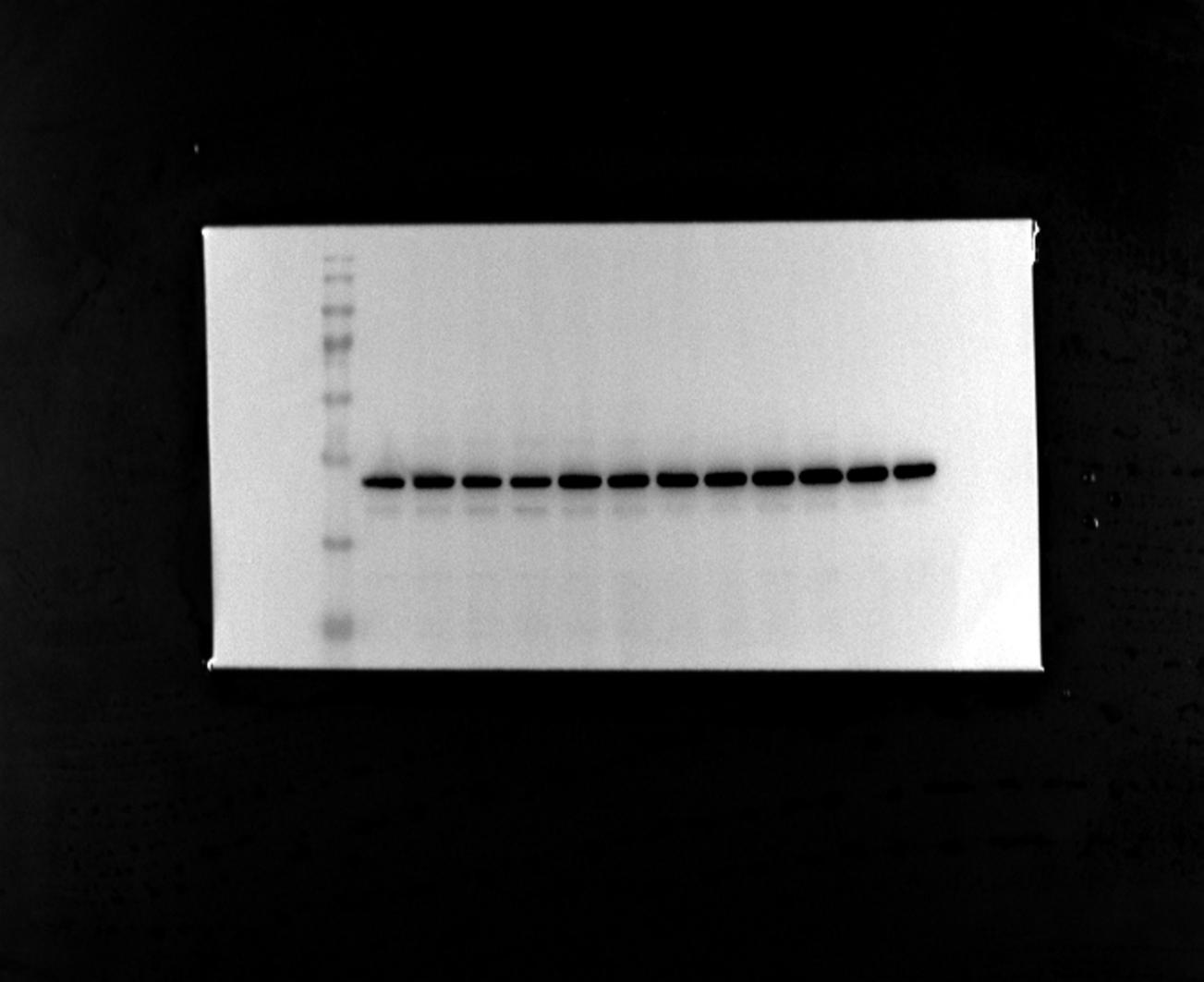


**N PPVL 4W 6W 8W 10W**

**45kD**

②

PAI-1

**N PPVL 4W 6W 8W 10W**


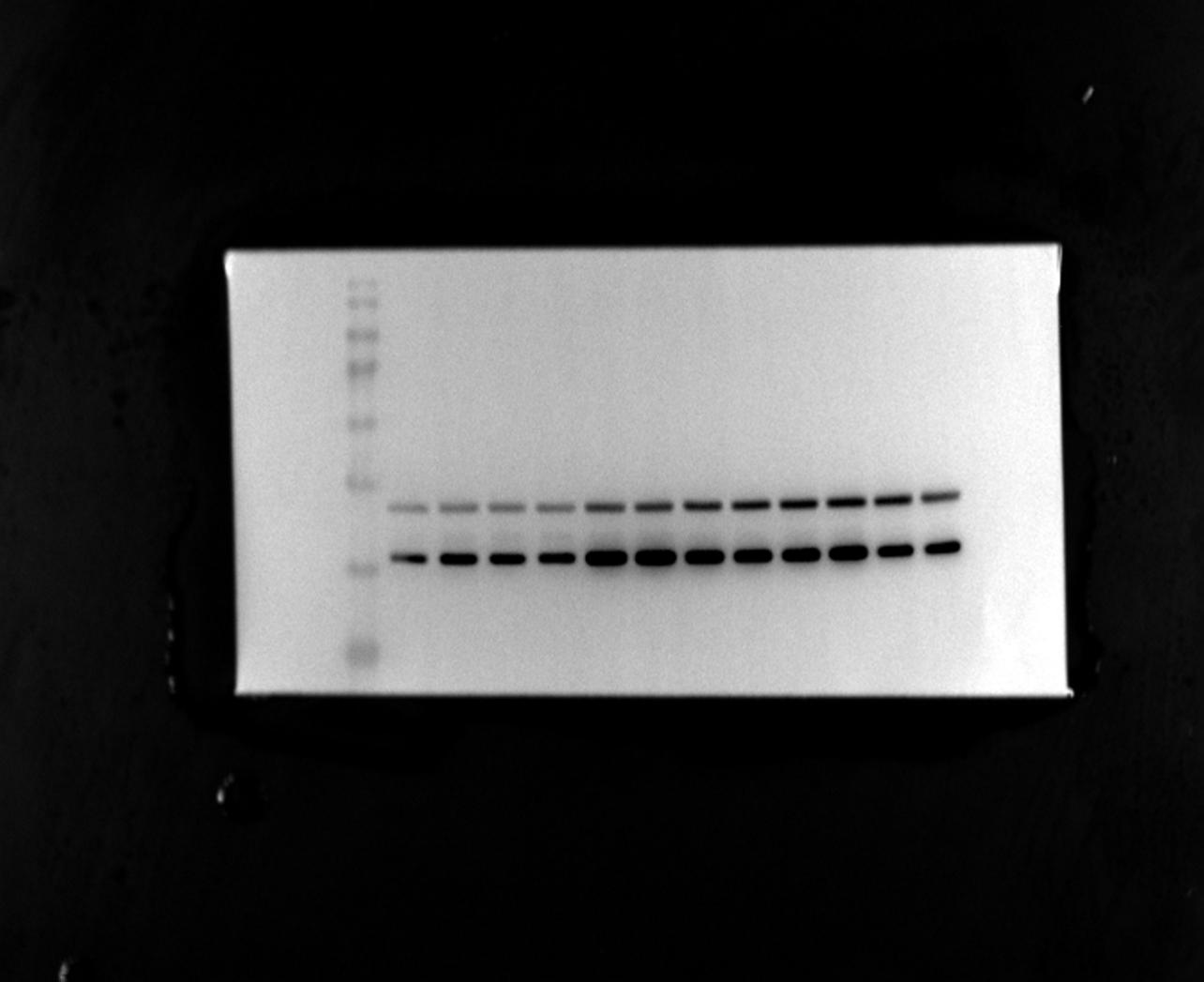


**N PPVL 4W 6W 8W 10W**

**N PPVL 4W 6W 8W 10W**

**37kD**

②GAPDH

Fibrin


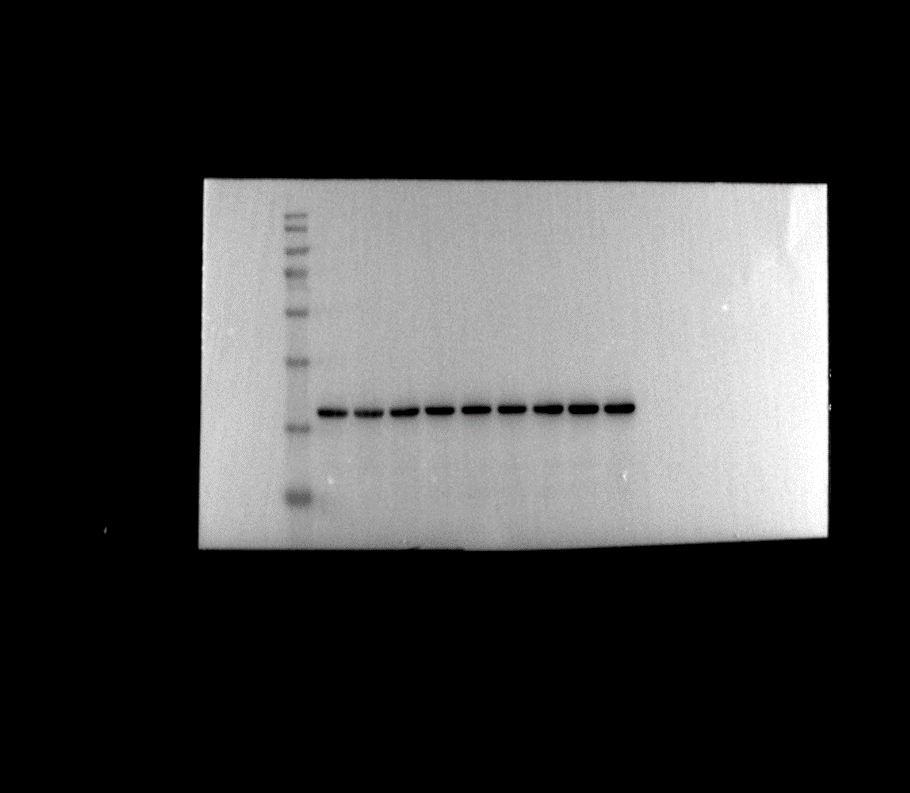

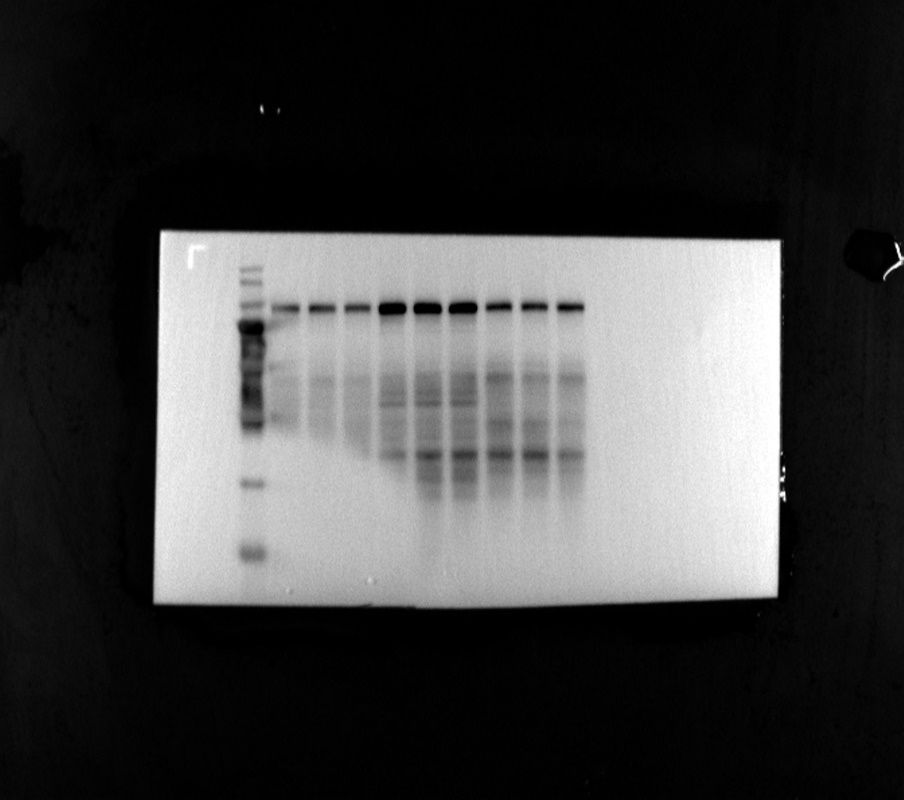


**95kD**

**N 10W RIVA**

GAPDH

**N 10W RIVA**

**37kD**

**The represetative bands for Fibrin and GAPDH shown in Figure 8-C.**


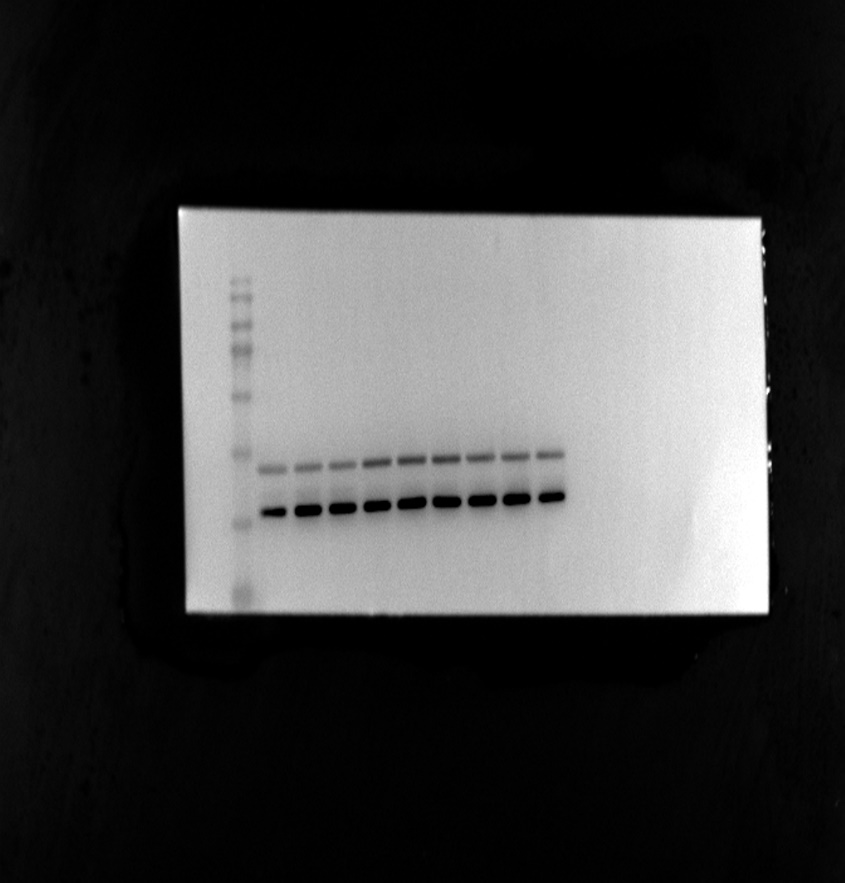

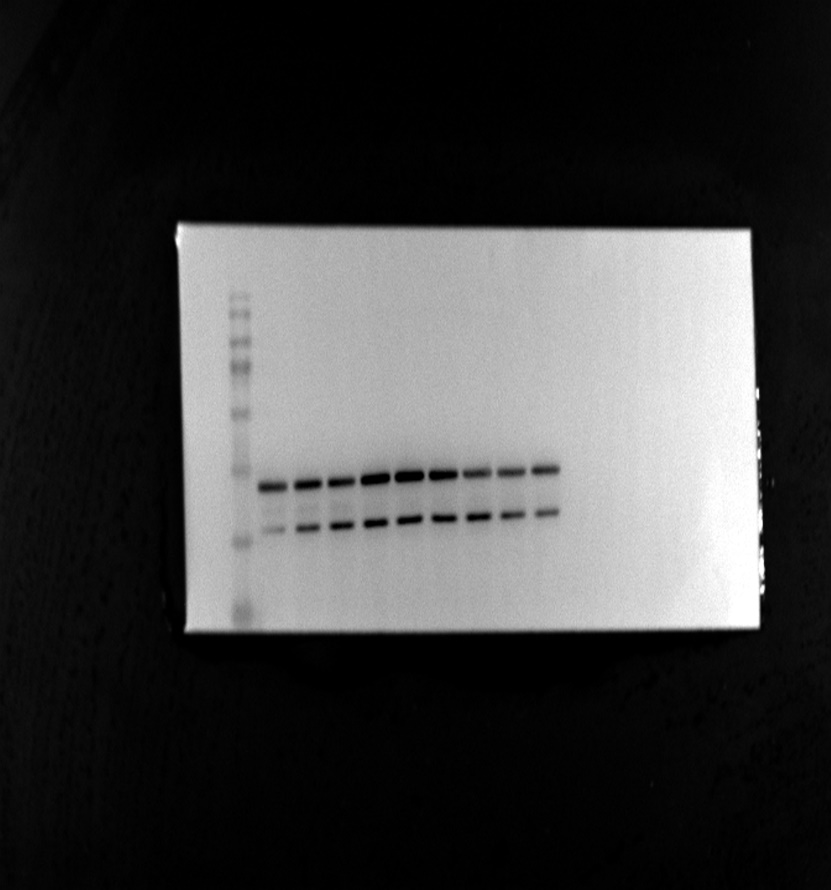


PAI-1

GAPDH

**45kD**

**N 10W RIVA**

**37kD**

**N 10W RIVA**

**The representative bands shown in Figure 8-C.**
